# Recent Trends of the Bio-Inspired Nanoparticles in Cancer Theranostics

**DOI:** 10.3389/fphar.2019.01264

**Published:** 2019-10-25

**Authors:** Vijay Sagar Madamsetty, Anubhab Mukherjee, Sudip Mukherjee

**Affiliations:** ^1^Department of Biochemistry and Molecular Biology, Mayo Clinic College of Medicine and Science, Jacksonville, FL, United States; ^2^Department of Formulation, Sealink Pharmaceuticals, Hyderabad, India; ^3^Department of Bioengineering, Rice University, Houston, TX, United States

**Keywords:** bio-inspired nanoparticles, cancer, theranostics, nanomedicine, imaging, clinical trials

## Abstract

In recent years, various nanomaterials have emerged as an exciting tool in cancer theranostic applications due to their multifunctional property and intrinsic molecular property aiding effective diagnosis, imaging, and successful therapy. However, chemically synthesized nanoparticles have several issues related to the cost, toxicity and effectiveness. In this context, bio-inspired nanoparticles (NPs) held edges over conventionally synthesized nanoparticles due to their low cost, easy synthesis and low toxicity. In this present review article, a detailed overview of the cancer theranostics applications of various bio-inspired has been provided. This includes the recent examples of liposomes, lipid nanoparticles, protein nanoparticles, inorganic nanoparticles, and viral nanoparticles. Finally, challenges and the future scopes of these NPs in cancer therapy and diagnostics applications are highlighted.

## Introduction

In recent years, prodigious progress of nanotechnology prompted researchers across the globe to ponder over the enormous possibilities of commissioning this for developing imaging and diagnostic-based medicine for cancer. Ergo, multimodal theranostics (therapy plus diagnostic) nanomedicine has emerged as a providential paradigm in cancer therapy (Lammers et al., 2011; Kelkar and Reineke, 2011; Muthuraj et al., 2015; Muthuraj et al., 2016; Chowdhury et al., 2017). It entails the benefits of both worlds: highly efficacious nanocarriers to ferry cargo while loading them onto both imaging and therapeutic agents. This buoyant proposition kindled legions of newly generated nanoparticles added in the tapestry of nanotechnology, having dual capacity of therapeutic delivery and diagnosis, which, in turn, hastened the preponderance of personalized medicine. It turns out that four crucial aspects exist to take into account while designing an efficient theranostic based nano-platform: i) Selecting an effective therapeutic agent, ii) to opt a stable carrier; iii) to implement a targeting and sustainable drug release approach; iv) to cautiously select an imaging agent (Mukherjee et al., 2014; Muthuraj et al., 2016; Chi et al., 2017; Chowdhury et al., 2017; Cong et al., 2018).

Nanocarriers, normatively being within the size range of 1 to 100 nm, have majorly been employed in biomedical applications (Meka et al., 2019; Mukherjee et al., 2019a). Among a phenomenally diverse range of nano-systems with their divergent synthetic routes, bio-inspired methods are appraised to be superior to chemical methods as the latter involves consumption of noxious materials resulting in abysmal consequences (Lin et al., 2012; Duan et al., 2015). Bio-nanoparticles, thus, have become part of the zeitgeist owing to their congenial physico-chemical properties. The most promising members in the cauldron are: viral NPs, protein NPs, apoferritn, aptamers, solid-lipid NPs, etc (Sivarajakumar et al., 2018; Mukherjee et al., 2019a). Nonetheless, biosynthesized multifunctional nanoparticles with noble metal centers encapsulating therapeutic and imaging agents were shown to possess theranostic activities against cancer (Duan et al., 2015; Ovais et al., 2018; Ma et al., 2018; Vemuri et al., 2019). In general, bioinspired theranostic agents can be generated, as it were, *via* the following strategies: i) screening of plant extracts for the synthesis of nanoparticles; ii) standardization of various physicochemical parameters for biosynthesis; iii) addition of therapeutic and imaging agents; iv) characterization of nanocarriers using analytical methods (Duan et al., 2015).

In this review, we will summarize the recent advancements in synthesis and bio-activity evaluation of an array of bio-inspired nanoparticles to circumvent the challenges of the conventional cancer therapy, contemporary clinical status and future directions.

## Cancer, Global Statistics, Conventional Therapy, Challenges, Alternative Approaches

Cancer remains the second leading threat to human survival in the world and was responsible for an anticipated 9.6 million deaths in 2018. Around one in six deaths worldwide is due to cancer. It turns out that ∼70% of deaths from cancer happen in low- and middle-income countries. It also turns out that five major lifestyle and food habit related issues are responsible for one third of deaths from cancer: i) use of tobacco, ii) high body mass index, iii) low fruit and vegetable intake, iv) lack of physical activity, and v) alcohol use. Among these, tobacco use has been proven to be most detrimental for cancer occurrence and causes ∼22% of cancer deaths. Viral infections, leading to cancer, are also accountable for egregious demise (∼25%) of human population in poverty-stricken countries. (Ferlay et al., 2015; Bray et al., 2018; Collaborators, 2018).

Conventional cancer therapy includes surgical intervention, chemotherapy, and radiation therapy, among which chemotherapy, individual and combinatorial, has remained the foremost modality for the treatment of cancer for the last several decades (Devita, 1978; Hanna and Einhorn, 2014). Afterwards, an enhanced understanding of cancer biology has engendered a new era of targeted cancer treatment by utilizing few inimitable properties of cancerous cells (Hanahan and Weinberg, 2011). In addition, tumor specific antigens (TSA) and tumor associated antigens (TAA) expressed by cancer cells have been consigned as targets for monoclonal antibody (mAb)-based therapy (Vigneron et al., 2013). Antibody-drug-conjugates (ADC) have also paved their way from bench-side to bed-side in a majestic way (Mukherjee et al., 2019b).

Despite these substantial progresses, each strategy suffers from some intrinsic limitations and thus scientists and researchers have shifted their focal point on the development of the nanoparticulate therapeutic systems, including liposomes, polymeric nanoparticles, lipid-polymer hybrids, metal nanoparticles, bio-nanoparticles, etc. The ability of nanosystems to specifically accumulate in tumor cells, i.e. EPR (enhanced permeability and retention) effect, is largely attributed to their small size and the leaky tumor vascularization. Furthermore, while carrying the freight of therapeutics onto them, these NPs can be reoriented as well as redirected in multiple ways (Mukherjee and Patra, 2016; Yue and Dai, 2018; Mukherjee et al., 2019c). Needless to say, bio-inspired nanoparticles have attracted an ample amount of research interest in last few years. In the following sections, we shall recapitulate the landmark progresses in their application as theranostics in cancer therapy.

## Nanomedicine in Cancer Theranostics

**Figure 1:**
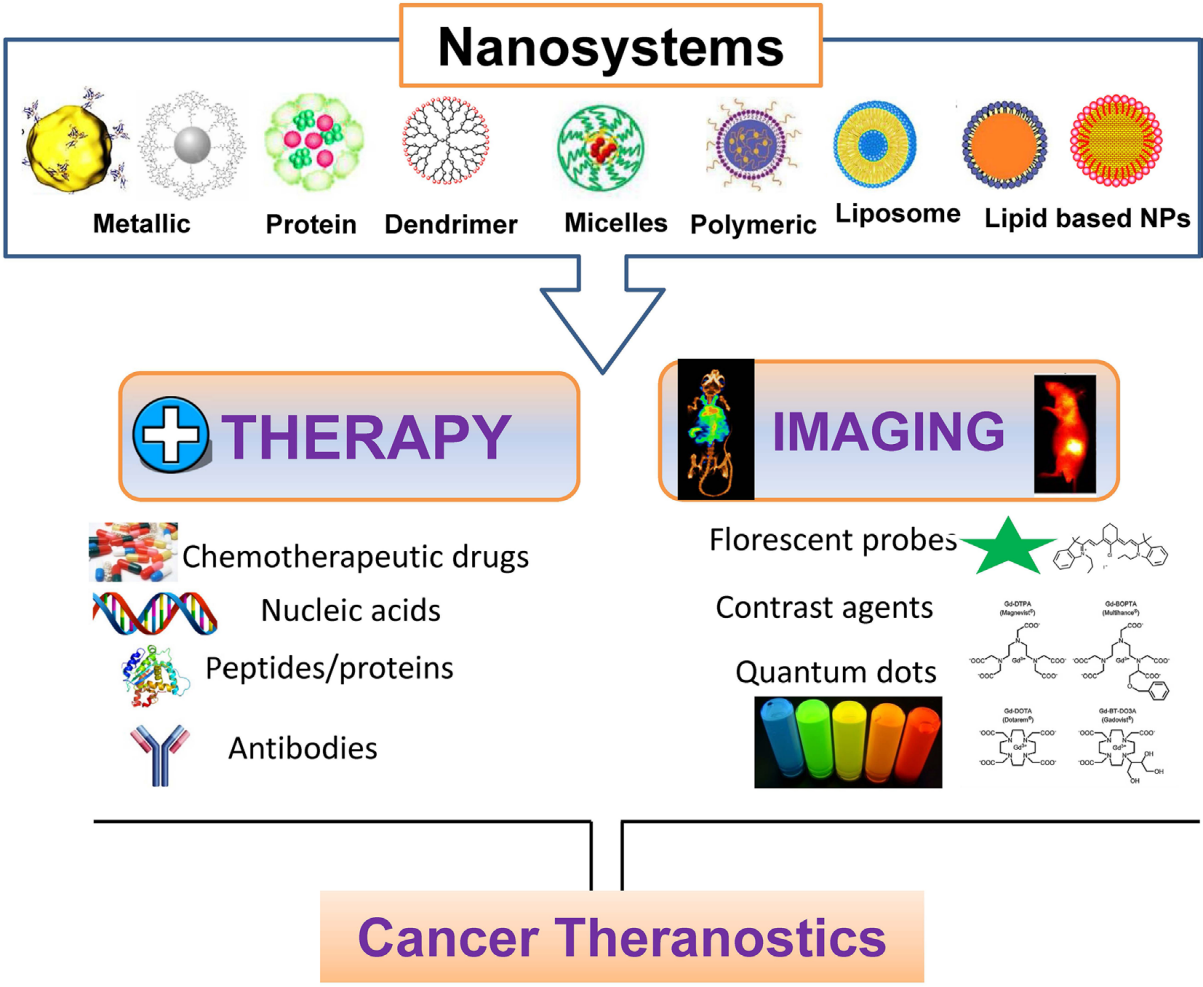
Nanotheranostics: polymeric, lipid-based and metallic nanomaterials for cancer theranostics.

Nanotechnology is one of the most rapidly growing fields in biomedical science, which has been smartly used to unravel various biological challenges (Mukherjee and Patra, 2016; Yue and Dai, 2018; Mukherjee et al., 2019c). Recently, nanotechnology has been vastly utilized for the diagnosis and treatment of many diseases including cardiovascular diseases, diabetes, cancer, bacterial infections, neuro-disease, etc. Owing to various above mentioned limitations in the conventional therapeutic strategies, different research groups have focused on developing nanoscale agents, including liposomal nanoparticles, metal nanoparticles, viral nanoparticles, protein nanoparticles and lipid nanoparticles (Mukherjee and Patra, 2016; Yue and Dai, 2018; Mukherjee et al., 2019c) (**Figure 1**). It is important to mention that nanoparticles have considerably improved the diagnostics and therapeutics of various cancers due to small size, ease of functionalization, enhanced drug loading (due to large surface to volume ratio), effortless penetration abilities, and improved retention inside target tissue. Apart from that, excellent biocompatibility, biodegradability, multifunctional applications including bio-imaging, bio-sensing, diagnostics and therapeutics, has increased the potential use of these nanomaterials for various biomedical applications. Here, we are summarizing several proof-of-concept applications of nanosystems, which are currently FDA approved or under clinical trials tabulated in **Table 1**. Although, currently the number of nanotheranostics agents in clinical trial is less, we believe that it will expand at a rapid rate very soon after watching their brisk progress.

**Table 1:** Nanotheranostic systems in a clinical stage of development.

Nanosystem	Description	Cancer type	Sponsor/Agency	Clinical Trial ID/Phase
Silica Nanoparticles	For Real-Time Imaging of Lymph node Metastasis	Colon, Head & Neck, Breast Cancer	Memorial Sloan Kettering Cancer Center	NCT02106598 Phase 1&2
Polysiloxane Gd-Nanoparticles	To assessing the MTD of AGuIX-NP along with cisplatin & radiation in cervical cancer patients.	advanced cervical cancer	Gustave Roussy, NCI, France	NCT03308604 Phase 1
Liposomes	Evaluating Immunogenic Chemotherapy Combined With Ipilimumab and Nivolumab in Patients With Metastatic Luminal B Breast Cancer	Breast Cancer	Oslo University Hospital	NCT03409198 Phase 2B
Carbon Nanoparticles	Use of carbon nanoparticles for primary and lymph nodes tumors isolation and mapping in the laparoscopic surgery.	Colorectal Tumor	Aiguo, Lu	NCT03350945 Not Applicable
Protein-based Nanoparticles	To perceive the side effects & MTD of EphB4-HSA fusion protein with chemotherapy in patients with solid tumors.	Solid Tumors	Vasgene Therapeutics, Inc	NCT02495896 Phase 1
hafnium oxide (HfO2) Nanoparticle	To find MTD of NBTXR3 which is, activated by radiation, Brachytherapy	Prostate cancer	Nanobiotix	NCT02805894 Phase 1
Polymeric Nanoparticles	To estimate MTD, safety, PK, PD of AZD2811 with/without azacitidine in patients with relapsed AML	Acute Myeloid Leukaemia	AstraZeneca	NCT03217838 Phase 1
Gold Nanoparticles	To estimate the efficacy of NU-0129, (nucleic acids prepared on the exterior of a spherical gold nanoparticle)	Glioblastoma	Northwestern University National Cancer Institute (NCI)	NCT03020017 Early Phase 1
Polymeric Nanoparticles	To study the anti-cancer effect of CetuximabNanoparticles.	Colon Cancer	Ahmed A. H. Abdellatif	NCT03774680 Phase 1
Polymeric Nanoparticles	Open-label PET study with [89Zr]-Df-CriPec^®^ docetaxel in patients with solid tumors.	Solid Tumor	Cristal Therapeutics VU University Medical Center	NCT03712423 Phase 1
Polymeric Nanoparticles	To evaluate antitumor activity CRLX101 plus enzalutamide in prostate cancer patients who already treated with enzalutamide.	Prostate cancer	National Cancer Institute (NCI)	NCT03531827 Phase 2
Micelles	To Evaluate the Efficacy and Safety of Docetaxel Micellein Recurrent or Metastatic HNSCC	Head & Neck Squamous Cell Carcinoma	Samyang Biopharmaceutical Corporation	NCT02639858 Phase 2
Liposomes	To study the distribution profile and radiation dosimetry of ^188^Re-BMEDAliposomes.	Tumors	Nuclear Energy ResearchInstitute of Taiwan.	NCT02271516 Phase 1
Liposomes	To study the MTD of EphA2 siRNA –encapsulated liposomes, evaluate efficacy in the tumor cell, which we cannot be cured by treatment.	Solid tumors	M.D. Anderson Cancer Center National Cancer Institute (NCI)	NCT02191878 Phase 3
Protein-Based Nanoparticles	To determine the MTD of ABI-009 and evaluate safety and anti-tumor activity.	bladder cancer	National Cancer Institute,Aadi, LLC	NCT02009332, Phase 1
Protein-Based Nanoparticles	To evaluate the activity of ONTAK in cutaneous T-cell Lymphoma (CTCL)	CTCL	Eisai Inc.TMC, Ligand pharmaceuticals,NCI.	NCT00211198 Phase 4
PSMA-targeted Polymeric Nanoparticles	A Study of BIND-014 efficacy in various Lung Cancer patients	Squamous Cell, NSCLC	BIND Therapeutics	NCT02283320 Phase 2
Protein-Based Nanoparticles	To find MTD for rapamycin loaded albumin with standard chemotherapy in solid tumors	solid tumors	National Cancer Institute Children’s Oncology group	NCT02975882 Phase 1
PSMA-conjugates	To determine the activity of Lu-PSMA vs cabazitaxel in prostate cancer	prostate cancer	ANUPCTG, ANSTO, PCFA, ARTnet, and Movember Foundation	NCT03392428 Phase 2
CCK2 receptor targeting ^111^In peptide conjugates	Radioactivity uptake of ^111^In-CP04 in tumor and other tissues	Thyroid Carcinoma	Paola Anna Erba	NCT03246659 Phase 1
67Cu-Peptide conjugates	MTD study of 64Cu-SARTATE	Neuroblastoma	Clarity Pharmaceuticals Ltd	NCT04023331 Phase 1&2
Iron oxide nanoparticles (SPIONs)	To find the feasibility of using SPIONs-Ferumoxytol in Magnetic Resonance Imaging analysis	Head & Neck Cancer	M.D. Anderson Cancer Center	NCT01895829 Phase 1
Lipid-based nanoparticles	To study proposes targeted delivery cytotoxic drugs, viaformulated LTSL activated by using focused ultrasound (FUS).	Liver Tumors	University of Oxford	NCT02181075 Phase1
Gold nanoparticles	To evaluate the PTT efficacy of PEGylated AuroShell suspension	Primary and Metastatic Lung Tumors	Nanospectra Biosciences, Inc.	NCT01679470

## Bio-Inspired Nanomaterials in Cancer Theranostics

In recent times, bio-inspired nanoparticles mimicking natural components in the body have gained immense attention due to their ability to serve as alternative biocompatible drug delivery systems in cancer theranostics (Ngandeu Neubi et al., 2018). The foremost advantage of these nanoparticles lies in the basic changes in systemic bio-distribution that non-native drug delivery systems don’t show. This present review will try to provide an inclusive understanding about different types of bio-inspired nanomaterials including liposomes, lipid nanoparticles, bio-synthesized metal nanoparticles, viral nanoparticles, protein nanoparticles, etc., towards cancertheranostics applications (Mukherjee et al., 2014; Evangelopoulos and Tasciotti, 2017; Ngandeu Neubi et al., 2018; Ovais et al., 2018; Zhang et al., 2018).

### Liposomes in Cancer Theranostics

Among the manifold lipid-based nano-arsenals available today, liposomes are definitely the most well recognized and versatile one due to their unique properties. They consist of unilamellar lipid bilayers that cocoon an aqueous core and offer numerous advantages, namely biocompatibility, biodegradability, ease of synthesis, sustained release of the therapeutics, low toxicity, and the ability to incorporate both hydrophilic and hydrophobic chemotherapeutic compounds. Their surfaces can also be modified for use in targeted cancer therapy (Silva et al., 2019). Due to several advantages a number of liposomal drugs are presently clinically approved and/or under clinical trials (Deshpande et al., 2013; Bozzuto and Molinari, 2015; Pattni et al., 2015; Lamichhane et al., 2018). Besides their ability to carry an array of small as well as large molecules, they have also been explored to deliver a myriad of diagnostic agents, including ^64^Cu (Petersen et al., 2012) & ^14^C isotopes (Al-Jamal et al., 2009), quantum dots (QDs) (Wang and Chao, 2018), gadolinium (Gd)-based contrast agents (Lamichhane et al., 2018), SPIONs (Martínez-González et al., 2016), and fluorescent probes (Portnoy et al., 2015; Shen et al., 2017; Lamichhane et al., 2018; Xing et al., 2018). Taken together, the potential of liposomes as a theranostic device in cancer is likely to be translated into clinical practice shortly.

### Lipid Nanoparticles (LNPs) in Cancer Theranostics

LNPs, owing to their biocompatibility and ease of scalability, remain one of the most lucrative platforms for cancer theranostics (Tang et al., 2018). For instance, Cyanine fluorescent dyes showed a capacity to convert light energy to heat energy upon NIR irradiation, which were used for both imaging and thermal ablation of tumor cells (Yi et al., 2014; Feng et al., 2016; Weber et al., 2016; Feng et al., 2017). Both the anticancer drug and imaging probe loaded LNPs also being established for treating solid tumors. DiR dyes exhibit elongated absorption wavelength, which results in condensed auto-fluorescence and enhanced tissue penetration for superior antitumor activity (Pansare et al., 2012). In another study, porphyrin loaded -LNPs were fabricated using apoE3 as a targeting ligand which displayed encouraging therapeutic efficacy in glioblastoma (Rajora et al., 2017). Importantly, Lin et al. developed a targeted LNPs co-loaded with NIR dye and siRNA which demonstrated highly sensitive NIR imaging, non-invasive and real-time monitoring of drug delivery and its response in an orthotopic prostate tumor model (Lin et al., 2014). Below are few sub-categories LNPs with their respective applications in cancer theranostics: (Lin et al., 2014; Feng et al., 2017; Hameed et al., 2018; Hua et al., 2018; Kang et al., 2018; Mendes et al., 2018; Tahmasbi Rad et al., 2019).

#### Solid Lipid Nanoparticles (SLNs)

SLNs are low-toxic spherical colloidal nanocarriers with average size ranging between 50 to 100 nm. Comprising of a solid lipid core (made up of fatty acids, triglycerides, etc.) alleviated by an interfacial surfactant layer, these particles were explored for use in cancer theranostics (Mehnert and Mäder, 2001; Souto et al., 2007; Mussi and Torchilin, 2013; Lopes et al., 2014). For example, Kuang and coworkers developed IR-780 iodide loaded tumor vasculature targeted SLNs to monitor PTT by imaging, which was selectively accumulated at glioblastoma tissue (Kuang et al., 2017). For interested readers, few articles are presented to cover the broad spectrum of SLNs and their use in cancer (Videira et al., 2002; Videira et al., 2006; Videira et al., 2012).

#### Nano-Structured Lipid

NLCs are composed of a mixture of liquid and solid lipids. The lipid matrix of NLCs can range from a defective crystalline to an amorphous structure, allowing high drug loading and sustained drug release properties when compared with other counterparts (Videira et al., 2002; Wissing et al., 2004; Videira et al., 2006; Videira et al., 2012). In 2017, Li et al. succeeded in developing a simple and multifunctional nanosystem of NIR dye loaded CXCR4-targeted NLCs. The nanosystem was able to impede tumor progression and prevent metastasis (Li et al., 2017). In another study, paclitaxel and quantum dots (QDs) dual-loaded NLCs were prepared which detected tumor by imaging and improved antitumor activity in murine tumor model of hepatocellular carcinoma (Olerile et al., 2017). Scientists envisage its clinical translation in coming years.

#### Lipid Nanocapsules (LNCs)

LNC, a next-generation biomimetic nano-system assuming a structure in between liposomes and polymeric NPs, can be prepared following phase inversion of emulsions and organic solvent-free based procedures (Huynh et al., 2009). Balzeau and team developed paclitaxel and DiD co-encapsulated LNCs containing NFL-TBS.40-63 peptide as targeting ligand for treating glioblastoma. These targeted LNCs preferentially accumulated by glioblastoma cells in mice (Balzeau et al., 2013). In other studies, LNCs were used for gene delivery like long-circulating DNA, so-called DNA-LNCs or plasmid DNA (Morille et al., 2010; David et al., 2013). Researchers hold an optimistic outlook for this platform as well.

#### Lipid-Based Micelles

Lipid-based micelles are spherical form of lipid molecules, which arranged themselves in aqueous solutions. These particles are promising nano-carriers for delivering anti-cancer agents, especially for water-insoluble chemotherapeutic drugs. Ma and his coworkers fabricated a lipid-based micelle and loaded docetaxel onto it (M-DOC), which exhibited superior therapeutic efficacy with low systemic toxicity in xenograft breast cancer model (Ma et al., 2012). They can also be exploited for cancer theranostics in future.

### Protein-Based Nanoparticles for Cancer Theranostics

Protein-based NPs have invoked high hopes in scientific minds for their natural availability and camaraderie with physiology. Following clinical approval of Abraxane (albumin-bound paclitaxel) by FDA, albumin has been considered as a potential carrier for delivering imaging/anticancer agents to tumor microenvironments (Rosenberg et al., 1990; Takakura et al., 1990; Lohcharoenkal et al., 2014). Abraxane allowed higher dosing than regular paclitaxel (Taxol) and patients showed a superior response rate augmenting progression-free survival with minimal toxicity (Gradishar, 2006; Miele et al., 2009). Albumin eventually has become a versatile delivery platform for low water soluble drugs like rapamycin (solubility in water is ∼2.5 mg·ml^−1^) (Gonzalez-Angulo et al., 2013). Albumin-bound rapamycin (ABI-009) underwent clinical trial for the treatment of non-hematologic malignancies. Numerous albumin-based NPs are presently in clinical trials (Gradishar et al., 2005; Gradishar, 2006; Ana et al., 2016; An and Zhang, 2017).

**Figure 2:**
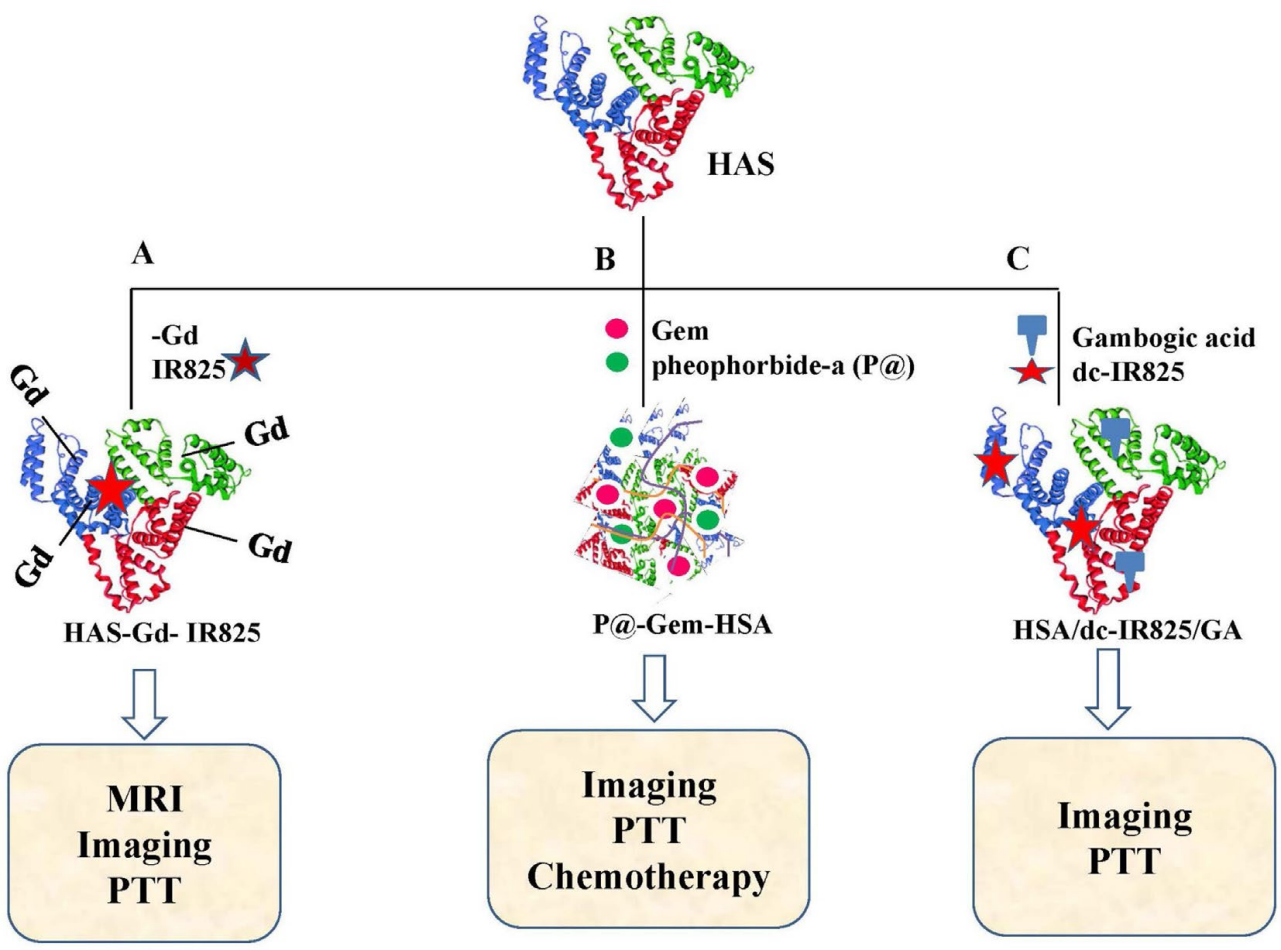
Schematic illustration of various HAS based nanoparticles. **(A)** HAS-Gd-IR825 (Chen et al., 2014), **(B)** P@-Gem-HSA (Yu et al., 2017), and **(C)** HSA/dc-IR825/GA complex (Gao et al., 2019).

Among other promising candidates, Human serum albumin (HSA) caps all since human liver provides a bountiful of the same (35 to 50 mg/ml). HSA has been explored as a natural transporter of inorganic/organic oxides, super-paramagnetic iron-oxide, IR825, IR780 and chlorin e6 (Ce6) for effective theranostics construction (Gou et al., 2018). Recent time has witnessed an emergence of a pull of NIR probes like indocyanine green (ICG), IR825 and IR780 for cancer theranostics as they have relatively deep tissue penetration capability and minimal auto-fluorescence interfering. Importantly, as mentioned in a current report, IR825 and Gadolinium (Gd) were enveloped in HSA to yield HAS-Gd-IR825 complexes (**Figure 2A**) for dual imaging-guided photothermal therapy (PTT) to inhibit lymphatic metastasis after surgery (Chen et al., 2014).

In another study, multifunctional nanoparticles were developed using gemcitabine and pheophorbide-a (P@) loaded human serum albumin (HSA) (P@-Gem-HSA, **Figure 2B**) for treatment of lymphatic metastases of PDAC (Yu et al., 2017). Most recently in 2019, Gao et al. established HSA NPs containing both heat shock protein (HSP90) inhibitor (gambogic acid, GA) and an imaging and photothermal agent (dc‐IR825) (**Figure 2C**) (Gao et al., 2019). These nano-formulations (HSA/dc‐IR825/GA) performed well in diagnosis and PTT mediated tumor growth inhibition. To date, several other protein-based NPs including ferritin, gelatin, Elastin, Gliadin, Legumin, transferring, Soy Proteins, Silk, Zein and milk proteins were developed and their efficacies in various preclinical models were evaluated. Articles summarizing their elaborate descriptions are being provided for interested readers (Podaralla and Perumal, 2010; Huang et al., 2014; Lohcharoenkal et al., 2014; Muthu et al., 2015; Yan et al., 2015; Liu et al., 2016; Tran et al., 2017; Wang et al., 2017a; Gou et al., 2018).

Apoferritin is a vacant protein nanocage made of self-assembling 24 polypeptide units without the iron core. Interestingly, apoferritin endures a process of assembly and disassembly by the change in pH, which has been majorly utilized towards the synthesis of different nanoparticles in cancer theranostics (Li et al., 2012; Zhao et al., 2016; Dostalova et al., 2017; Wang et al., 2017b). Luo et al., reported the synthesis of hyaluronic acid (HA)-conjugated apoferritin nanocages for pH-responsive controlled delivery of daunomycin (DN), encapsulated into the core of apoferritin (Luo et al., 2015). The apoferritin was conjugated to HA that has been used for the selective targeting and killing the cancer cells upon binding to the CD44 receptor. Another recent report by Li et al. showed the diagnosis of lung cancer using MR and fluorescence imaging of a multifunctional apoferritin nanostructure (Li et al., 2012). The authors developed a multifunctional hybrid nanostructure of ferritin that demonstrated a strong green fluorescence. Moreover, the presence of ferrimagnetic iron oxide nanoparticles into the hollow ferritin cavity was critical for the MR imaging of αvβ3 integrin upregulated cancer cells. Liang et al, utilized a biocompatible H-ferritin (HFn) nanocarrier for the targeted delivery of doxorubicin (Dox) to cancerous cells causing significant inhibition of tumor growth with a single-dose treatment in various subcutaneous murine cancer models (HT-29, A375, or MDA-MB-231) (Liang et al., 2014).

### Viral Nanoparticles in Cancer Theranostics

Viruses, the most resilient parasites, are largely explored in delivering therapeutics for their natural availability and prodigality of reproduction which, in turn, aid in scalability (Resch-Genger et al., 2008; Steinmetz, 2010; Yildiz et al., 2011; Czapar and Steinmetz, 2017). Virus-like particles (VLPs), a sub-category of VNPs, manifest innocuous demeanor due to a deficit in genomic nucleic acid (Plummer and Manchester, 2011; Yildiz et al., 2011). VNPs can be re-engineered with the targeted ligand, imaging reagents, and chemotherapeutic drugs (Au - Wen et al., 2012). For example, fluorescent CPMV sensors allowed for the visualization of the flow of blood and vasculature in living chick-embryos up to 500 μm depths, which has been further utilized in tumor angiogenesis imaging (Lewis et al., 2006; Suci et al., 2007). Importantly, Rosenberg and fellow-workers, back in 1990, conducted a retroviral gene therapy clinical trial with patients who already had reached an advanced stage of melanoma (Rosenberg et al., 1990). Recently, VNPs are also recognized as components for non-invasive imaging applications, namely, PET and MRI (Datta et al., 2008; Shukla and Steinmetz, 2015). For instance, ^18^F-fluoride and iron oxide particles were loaded into packets resulting from Hemagglutinating virus of Japan (HVJ) and accomplished high signal-to-noise imaging resolution in PET (Flexman et al., 2008). Later, several clinical trials were performed based on VLPs, and some were used in the clinical ready. Taken together, VNPs and VLPs provide the foundation for a various range of biomedical applications, including disease prevention, diagnosis, monitoring, and therapy.

### Inorganic Nanoparticles in Cancer

Inorganic nanoparticles including gold, silver, silica, rare earth oxides, iron oxides, and zinc oxide have been extensively used for several biomedical applications including cancer theranostics, bio-sensing, bio-imaging, drug and nucleic acid delivery due to their unusual physico-chemical properties (Patra et al., 2014; Lim et al., 2015; Gao et al., 2017; Gaddam et al., 2017; Silva et al., 2019; Mukherjee et al., 2019a). Recently, bio-synthesis of inorganic nanoparticles using green chemistry approach have gained immense attention due to many advantages over conventional chemical synthesis methods including (i) simple, fast and inexpensive method, (ii) eco-friendly approach due to avoidance of toxic chemicals, (iii) easy availability of large pool of bio-reducing agents including algae, plants, bacteria etc, (iv) use of commonly tolerable solvent i.e. water. Consequently, bio-synthesized inorganic NPs were utilized for several biomedical applications including cancer theranostics. Many research groups including ours demonstrated cancer theranostics application of biosynthesized metal nanoparticles including gold (AuNPs) and silver (AgNPs) nanoparticles.

Bio-synthesized AuNPs and AgNPs showed potential application in the delivery of anti-cancer drugs *in vitro* and *in vivo* (Patra et al., 2015; Mukherjee et al., 2016). Mukherjee et al. demonstrated the delivery of doxorubicin (Dox) using b-AuNPs synthesized using the aqueous leaf extract of Peltophorum pterocarpum, the ‘yellow flame tree’ towards *in vitro* and *in vivo* murine melanoma tumor model. Importantly, these b-AuNPs showed excellent biocompatibility when the C57BL6/J mice were treated with b-AuNPs compared to the mice treated with chemically synthesized AuNPs after seven consecutive intraperitoneal injections of 10 mg/kg/b.w. of dose (Mukherjee et al., 2016). Moreover, the b-AuNPs conjugated Dox treatment caused a significant reduction of melanoma tumor growth in mice compared to pristine Dox. In another published report by Ganeshkumar et al. the anti-cancer drug 5-fluorouracil was delivered using folic acid conjugated b-AuNPs (synthesized using the fruit peel extract of Punica granatum) in breast cancer cells in a targeted manner (Ganeshkumar et al., 2013).

**Figure 3:**
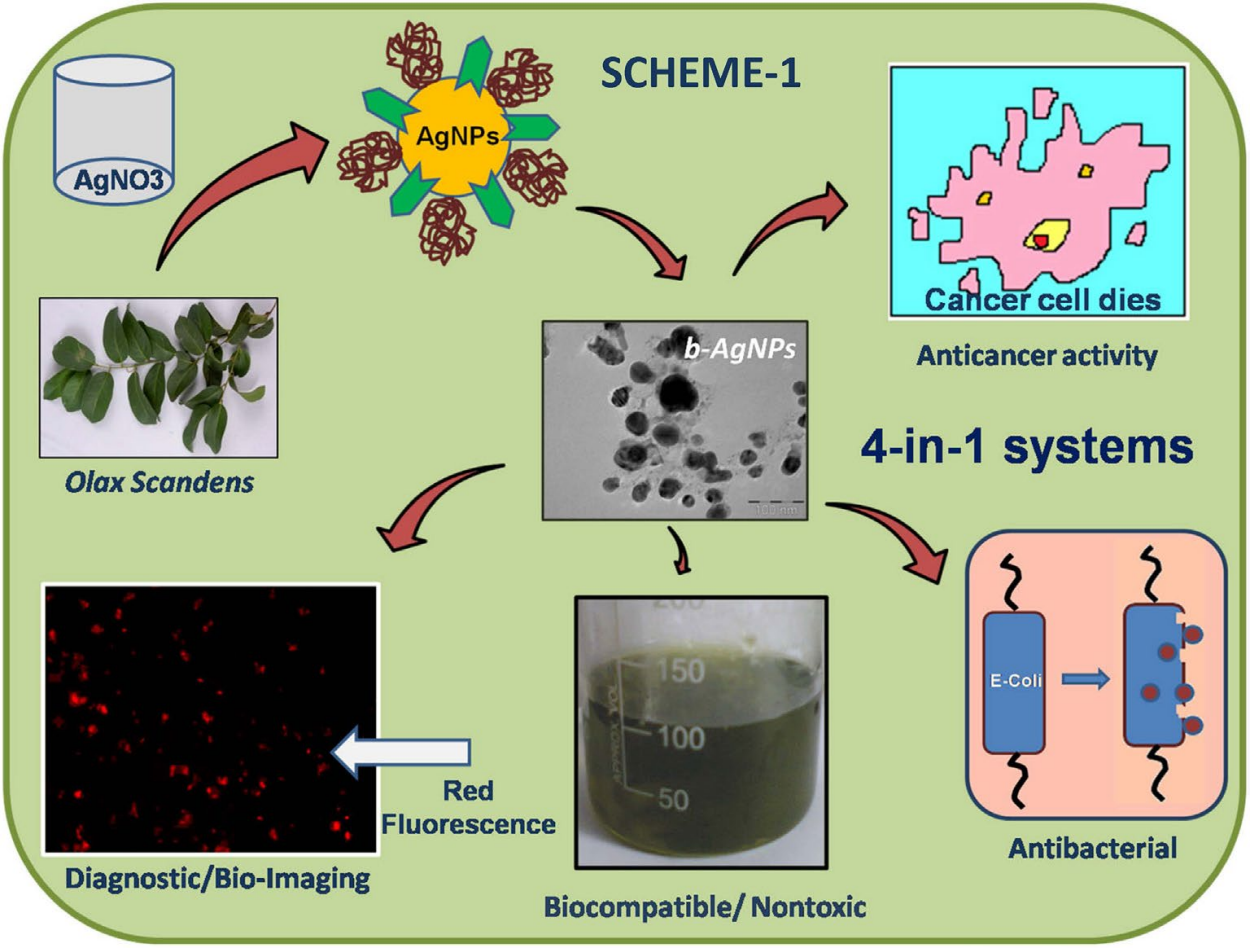
Over all presentation for synthesis, characterization and biomedical applications (diagnostic, anticancer antibacterial applications) of biosynthesized silver nanoparticles (b-AgNPs) using *Olax Scandens* leaf extract. Reprinted with permission from (Mukherjee et al., 2014).

**Figure 4:**
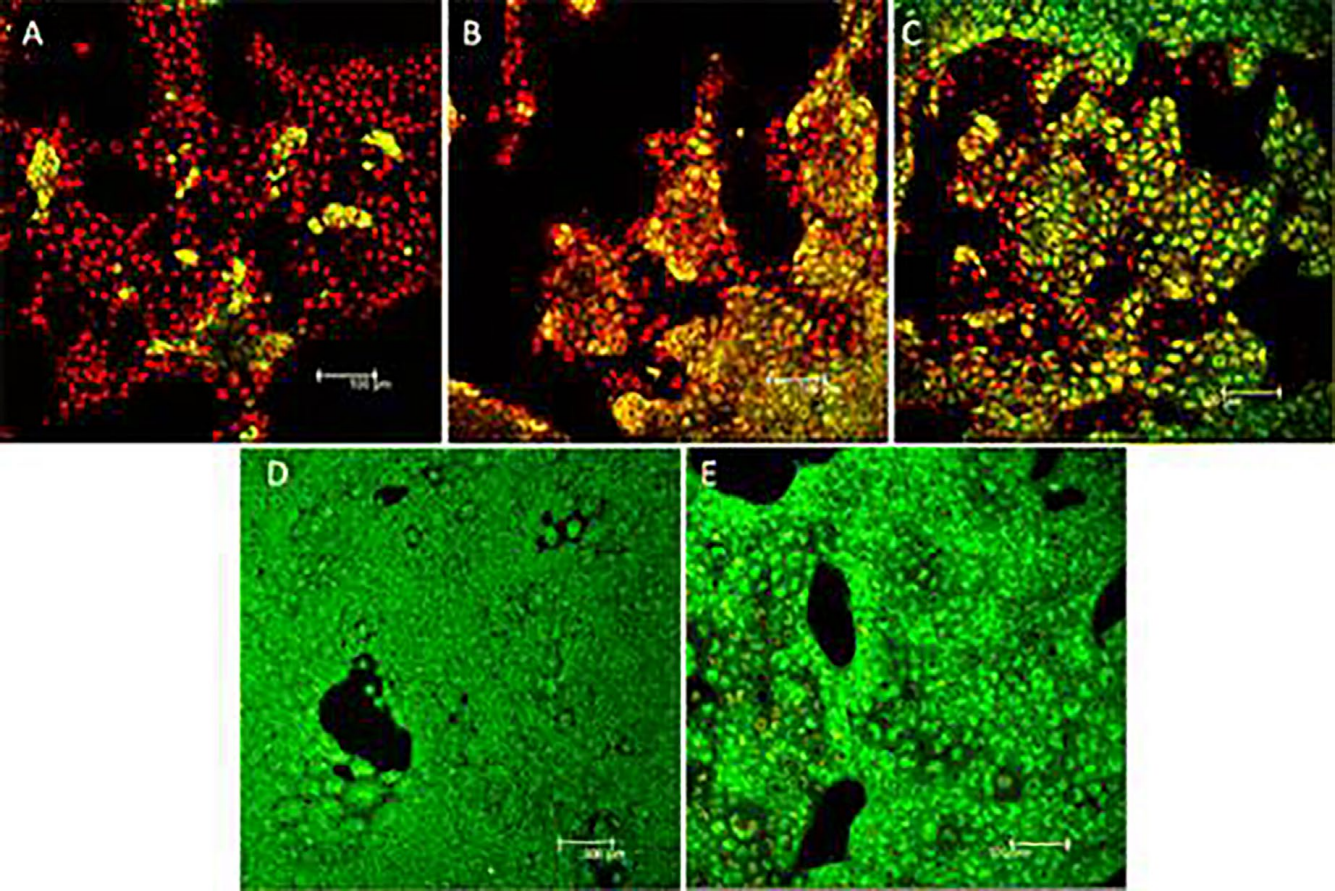
Live/dead staining of A431 cells after 800 nm laser irradiation for 10 min at power = 36 mW. Nanoparticle concentration in panels **(A**–**C)** and **(D)** are 200, 100, 50, and 0 μg/ml respectively. Decreasing amounts of red fluorescence is obtained with lower concentration of nanoparticles which indicates the photothermal activity of the nanoparticles in cell killing. **(E)** Live/dead staining of A431 cells treated with 100 μg/ml nanoparticle concentration without laser treatment. Reprinted with permission from (Fazal et al., 2014). Copyright @ American Chemical Society.

These b-NPs were utilized as anti-cancer agents due to the presence of therapeutically active phytochemicals (flavonoids, taxol, polyphenols and isoflavons) in the bioresources that attaches over the nano-surface through the biosynthesis process. Therefore, b-NPs display a distinctive advantage over chemically synthesized NPs. Mukherjee et al, showed the 4-in-1 theranostics applications (anticancer, biocompatible, antibacterial, and cell imaging) of bio-synthesized silver nanoparticles using methanolic extract of *Olax scandens* leaves (Mukherjee et al., 2014) (**Figure 3**). Mukherjee et al, demonstrated the anticancer activity of b-AuNPs prepared using the leaf extract of *Lantana montevidensis* (Mukherjee et al., 2015). Moreover, the leaf extract of *Lantana montevidensis* has several anti-cancer phytochemicals including apigenin, cirsilineol, hispidulin, eupatorine, β-caryophyllene and eupafolin responsible for their therapeutic efficacy. Fazal et al. reported photothermal ablation activities of anisotropic b-AuNPs synthesized using seeds extract of *Theobroma cacao* [cocoa] towards *in vitro* epidermoid carcinoma A431 cells upon laser exposure (Fazal et al., 2014) (**Figure 4**).

In another published report by Wang et al. the authors exhibited the fluorescence based bio-imaging of *in situ* b-AuNPs for the detection of tumors (Wang et al., 2013). Human leukemia cells (K562) and human hepatocarcinoma cells (HepG2) were incubated with chloroauric acid (HAuCl_4_) that showed green fluorescence whereas non-cancerous L02 (human embryo liver cell strand) cells didn’t show any fluorescence after incubation with HAuCl_4_ indicating the specificity of b-AuNPs synthesis to cancerous cells. The authors further showed the *in vivo* bio-imaging application towards *in vivo* xenograft tumor model of HepG2 or K562 cancer cells in BALB/c mice followed by subcutaneous injection of HAuCl_4_ solution (10 mmol/L). The *in vivo* fluorescence demonstrated bright fluorescence around the tumor even after 72 h of HAuCl_4_ injection indicating the constant fluorescence of *in vivo* bio-synthesized b-AuNPs, which can be utilized for the diagnosis of tumor.

## Future Perspective and Conclusive Remarks

The nanomaterials and their application in cancer theranostics has been moving ahead over the last several years but many challenges restrain its clinical translation. One of the toughest challenges in the nano-theranostic field is to have multiple functionalities planted into a single NP making the system complicated. The adversity is reflected during the process of industrial scale up production and future clinical translation. Scientists and engineers in the field need to act upon this. Successful application of cancer theranostics nanomedicine depends on choosing the appropriate imaging and contrast modalities for the exact clinical circumstance. Hence, an interdisciplinary approach should be firmly followed in order to facilitate the use of the nanomaterials towards cancer theranostics. Firstly, the regulatory agencies should modernize the guidelines for these nano-theranostic platforms in order to assist for the assessment of efficacy, safety and, speeding up their clinical translation. Next, for those who research and develop these nanoplatform agents, it might be challenging to simultaneously optimize dose levels and administration frequencies using a single delivery platform. Drug encapsulation optimization, ligand conjugation efficiency, and high reproducibility with low cost biomaterials are important for future clinical application of nano-theranostics. Mechanistic investigations of the interaction of the NPs with proteins, lipids and immune system and their subsequent clearance from the human body should be pursued in detail. Moreover, considerable *in vivo* assessment is of significance and highly required. Finally, further development of novel nano-theranostics agents is required to allow high-resolution imaging and to mitigate the background of common techniques including the fluorescence and other limitations of photo-bleaching. In conclusion, with many encouraging evidences sprouted in recent years where new drug delivery and imaging technologies are developed, there is a positive outlook for bio-inspired nano-theranostics and its clinical translation could be realized in the near future to transfigure cancer therapy.

## Author Contributions

Conceptualization: AM and SM. Writing—Original Draft Preparation: AM, VM, and SM. Writing—Review and Editing: AM, VM, and SM. Funding Acquisition: SM.

## Funding

This research received no external funding. The APC was partially funded by Fondren Library, Rice University, Houston, TX, USA.

## Conflict of Interest

Anubhab Mukherjee was employed by Sealink Pharmaceuticals Pvt Ltd.

The remaining authors declare that the research was conducted in the absence of any commercial or financial relationships that could be construed as a potential conflict of interest. The authors have no other pertinent affiliations or financial connection with any organization or entity with a financial interest in or financial conflict with the subject matter or materials discussed in the manuscript apart from those disclosed.
